# Paving the Way for ERAS in German Gynecologic and Gynecologic Oncology Departments: Insights into Barriers, Facilitators and Practical Strategies

**DOI:** 10.3390/healthcare14050682

**Published:** 2026-03-08

**Authors:** Cara Thiel, Helena Schorling, Lina Judit Schiestl, Mona Wanda Schmidt, Anne-Sophie Heimes, Kathrin Stewen, Gilbert Georg Klamminger, Lea Omogbehin, Katharina Delfs, Konstantin Hofmann, Evangelos Papanikolaou, Georgios Tagarakis, Ioannis Boutas, Annette Hasenburg, Roxana Schwab

**Affiliations:** 1 Department of Obstetrics and Gynecology, University Medical Center of the Johannes Gutenberg University Mainz, 55131 Mainz, Germany; cara.thiel@unimedizin-mainz.de (C.T.); helena.schorling@unimedizin-mainz.de (H.S.); lina.schiestl@unimedizin-mainz.de (L.J.S.); mona.schmidt@unimedizin-mainz.de (M.W.S.); anne-sophie.heimes@unimedizin-mainz.de (A.-S.H.); kathrin.stewen@unimedizin-mainz.de (K.S.); katahrina.delfs@unimedizin-mainz.de (K.D.); annette.hasenburg@unimedizin-mainz.de (A.H.); 2 Obstetrics and Gynecology, Medical School, Faculty of Health Sciences, Aristotle University of Thessaloniki, 54636 Thessaloniki, Greece; evpapanikolaou@auth.gr; 3 Cardiothoracic Surgery, Medical School, Faculty of Health Sciences, Aristotle University of Thessaloniki, 54636 Thessaloniki, Greece; gtagarakis@gmail.com; 4 Breast Unit, Rea Maternity Hospital, 17564 Athens, Greece; drboutas@gmail.com

**Keywords:** Enhanced Recovery After Surgery (ERAS), implementation barriers, implementation facilitators, German gynecological departments, gynecologic oncology, perioperative care pathways, professional satisfaction, knowledge translation

## Abstract

**Highlights:**

**What are the main findings?**

- Limited ERAS knowledge and insufficient personnel identified as primary implementation barriers.
- Patient-centered benefits and interactive education emerged as the strongest facilitators.

**What are the implications of the main findings?**

- Targeted education and multidisciplinary support can substantially improve ERAS uptake.
- Strengthening ERAS knowledge may enhance professional satisfaction and staff engagement.

**Abstract:**

**Background:** Enhanced Recovery After Surgery (ERAS) protocols improve postoperative outcomes and promote multidisciplinary, evidence-based perioperative care. However, ERAS adoption in gynecological departments remains inconsistent, and the underlying implementation challenges are poorly understood. **Objective:** To identify key barriers, facilitators, and preferred implementation strategies influencing ERAS adoption in German gynecological departments, and to assess whether clinicians’ ERAS knowledge or institutional certification shapes these perceptions. **Methods:** We conducted a nationwide, web-based cross-sectional survey of gynecologic clinicians in Germany. The questionnaire assessed ERAS-related knowledge, current implementation status, and perceived barriers, facilitators, and strategies. Statistical analyses included equality of proportions tests, logistic regression, and internal consistency measurement. **Results:** A total of 116 clinicians participated; 66 provided data on barriers and 64 on facilitators and strategies. Only 37.9% reported routine ERAS use. The most frequently identified barriers were limited ERAS knowledge (40.9% “very important”) and insufficient personnel resources (40.9%). The strongest facilitators were improved patient well-being, reduced morbidity, and higher patient satisfaction (each >60% “very important”). High-impact implementation strategies included informational materials, workshops, and online training. Well-informed clinicians had significantly higher odds of reporting a positive professional impact of ERAS (OR = 9.0, *p* = 0.001). **Conclusions:** ERAS implementation in gynecological settings remains restricted by staff knowledge gaps and personnel limitations. Patient-centered benefits and interactive educational strategies serve as powerful facilitators. Enhanced staff education and multidisciplinary support structures may substantially improve ERAS uptake and contribute to greater professional satisfaction among clinicians.

## 1. Introduction

Enhanced Recovery After Surgery (ERAS) is an evidence-based perioperative care approach designed to reduce surgical stress, improve recovery, and decrease postoperative complications [1,2,3,4]. In gynecologic and particularly gynecologic oncology surgery, ERAS Society guidelines have demonstrated significant benefits, including reduced morbidity and shorter hospital stay across open and minimally invasive procedures [5,6,7,8]. Despite this evidence base, implementation remains inconsistent, and adherence to recommended elements varies widely across institutions [8,9,10,11,12]. Although ERAS protocols have shown substantial benefits in improving recovery and reducing complications, their implementation continues to face widespread barriers [13]. Studies have consistently identified provider-related challenges, such as a lack of agreement with specific recommendations, resistance to changing established practices, limited motivation, and insufficient interdisciplinary communication, particularly between surgeons, nurses, and anaesthesiologists, as major players in protocol adherence [13,14,15,16]. Successful implementation often requires a fundamental shift in team culture, which can be difficult to achieve in systems resistant to change [9]. At the organizational level, barriers frequently include staff shortages, time constraints, lack of dedicated funding, and limited administrative or leadership support [13,14,17]. These resource-related challenges are further exacerbated by high staff turnover and the absence of institutional incentives or coordination frameworks [14]. Patient-related obstacles have also been reported, including variability in recovery, low compliance, and cultural beliefs [14].

Despite these barriers, several facilitators have been identified, including improved clinical outcomes, higher patient satisfaction, structured staff education, regular interdisciplinary meetings, and the presence of a dedicated ERAS coordinator [12,13,14,15,16,17,18]. Although previous studies have examined ERAS implementation in various surgical specialties and international settings, perception-based insights from gynecologic clinicians within the German healthcare context remain scarce. This highlights a critical evidence–practice gap: although ERAS is well supported by evidence, implementation in gynecologic care remains inconsistent, and the practical reasons for this are not yet fully understood [8,9,10,11,12,13,14,15,16,17,18,19].

While clinical guidelines provide the evidence base for ERAS, successful implementation depends equally on non-clinical factors such as institutional culture, staff engagement, and strategic leadership. Studies have shown that ERAS adoption is not a static event but a gradual process of adjustment, acceptance, and normalization, influenced by evolving staff attitudes and team experiences [9]. Key enablers include strong multidisciplinary collaboration, dedicated ERAS champions, and clearly defined team roles, which together foster both ownership and accountability [12,20,21]. Moreover, staff education, before and after implementation, is essential, particularly in settings with frequent personnel turnover, highlighting the need for sustained mentoring and structured feedback systems [12]. Importantly, effective communication, both within the team and with patients, has been linked to improved adherence and program sustainability [9,10,12,22].

Despite the increasing publication of ERAS recommendations in gynecologic oncology, there are currently no structured national data on implementation patterns in German gynecologic departments. The German hospital system, characterized by decentralized oncologic surgery and heterogeneous certification status, may influence ERAS uptake differently compared to more centralized systems.

This study aimed to identify the key barriers, facilitators, and practical strategies influencing the implementation of ERAS protocols in gynecological departments across Germany. A further aim was to explore whether institutional characteristics, specifically certification as a gynecologic oncology center (DKG), and clinicians’ familiarity with ERAS principles influenced the perception of these factors. While ERAS benefits are well established, perception-based evidence from the German gynecologic field remains limited. By collecting clinicians’ experiences and views, this study provides a context-specific perspective on implementation dynamics and highlights not only clinical factors but also the influence of ERAS on professional satisfaction, a dimension rarely explored in previous ERAS research.

## 2. Materials and Methods

### 2.1. Study Questionnaire

For the purpose of this study, a questionnaire was developed. Item generation was based on ERAS Society guidelines and previous ERAS implementation surveys. The final questionnaire covered prehabilitation, pre-, intra-, and postoperative ERAS measures, as well as barriers, facilitators, and implementation strategies (Supplementary Materials. Although the questionnaire was not formally based on a specific implementation framework, items were conceptually grouped into barriers, facilitators, and strategies following established implementation science principles.

Email invitations were sent to institutional departmental addresses. The sampling frame, therefore, consisted of all identifiable German gynecologic departments, ensuring broad national coverage [23]. No randomization or stratification was applied, reflecting a census-style distribution strategy.

No postal mail was used. A reminder email was distributed two and six weeks after the initial invitation to improve response rates. These intervals were selected based on standard survey-methodology recommendations to optimize response without inducing respondent fatigue.

German physicians aged over 18 years were eligible to participate. All participants provided informed consent before beginning the survey. Although the survey was conducted anonymously and did not collect identifiable personal data, participants explicitly confirmed their voluntary participation and consented to the publication of anonymized results, in accordance with principles of good ethical research practice.

The present analysis focuses exclusively on questionnaire items addressing implementation barriers, facilitators, and strategies. Clinical ERAS components (prehabilitation, intraoperative and postoperative measures) will be analyzed in a separate, pre-planned publication due to the extensive scope of the dataset and differing analytical aims.

### 2.2. Statistics

The statistical analysis was performed with R Studio, version 4.3.0 (2023-04-21 ucrt) (Copyright (C) 2023 The R Foundation for Statistical Computing).

No formal sample size calculation was performed because this study was designed as an exploratory, descriptive survey aimed at hypothesis generation. Also, subgroup and multivariable analyses must be interpreted cautiously and are considered exploratory. Additionally, no center identifiers were collected in order to preserve anonymity. Therefore, it was not possible to exclude multiple responses from the same institution or individual. This represents a potential source of clustering and overrepresentation.

Internal consistency of independent blocks of the questionnaire, such as “Barriers to implementation of ERAS”, was calculated using Cronbach’s alpha. A value above 0.7 was considered acceptable, and above 0.9 was considered excellent, indicating high reliability.

Frequency tables showed the distribution of responses for the various variables. The two-sample test for equality of proportions with continuity correction (Pearson’s chi-square test for equality of proportions) as implemented in the prop.test function in R was employed to calculate significant differences in proportions between factors considered very important or unimportant compared to the other factors. Where expected cell counts were small, we used Fisher’s exact test instead. Group-level analyses contrasted pooled response counts for the focal group of items (e.g., the four most frequently “very important” facilitators or the three most frequently “unimportant” factors) against pooled counts for all remaining items. Individual item analyses compared each focal item separately with the pooled remaining items. Because multiple focal items were tested in parallel, *p*-values from individual item comparisons were adjusted using the Benjamini–Hochberg procedure [24] to control the false discovery rate. All tests were two-sided except where a directed hypothesis was specified (i.e., that focal items would have a higher proportion of “very important” or “unimportant” ratings than the remaining items), in which case one-sided tests were used. A *p*-value < 0.05 was considered statistically significant.

As appropriate, exploratory logistic regression (for binomial factors) and ordinal regression (for ordinal variables) were employed to assess the potential impact/statistical significance of feeling well-informed about ERAS measurements or working in a certified DKG center. Results should be interpreted cautiously due to limited event numbers. For this purpose, the original four-level ERAS knowledge variable (“poor,” “somewhat poor,” “somewhat well,” “very well-informed”) was dichotomized into “poorly informed” (poor + somewhat poor) and “well-informed” (somewhat well + very well). This approach was chosen due to low cell counts in some categories, which limited the feasibility of ordinal regression and risked unstable model estimates. Positive professional impact” was defined as self-reported improvement in daily workflow or professional satisfaction as indicated by binary survey response (“positive influence” vs. “no influence”).

The odds ratio (OR) was used to determine if there was a trend between the independent variable and higher scores of the dependent variable. OR > 1 suggested a positive association, OR < 1 suggested a negative association. *p*-values < 0.05 were considered significant. Confidence intervals (CI) of 95% provided the range in which the true OR was expected.

## 3. Results

### 3.1. Demographic Characteristics of the Study Population

The web-based survey was conducted from 4 June to 18 September 2024. During this period, 420 access attempts were recorded; this number may include multiple entries from the same individual and therefore does not represent a count of unique invitees or unique survey initiations. Because the exact denominator of eligible clinicians is unknown and duplicate accesses cannot be excluded, a precise response rate cannot be calculated. The achieved sample size represents only a small fraction of German gynecologic clinicians and may not be representative. Consequently, the calculated proportion of respondents (116 valid responses) does not reflect a conventional response rate and cannot be used to precisely assess non-response bias.

Responses were considered valid if at least the demographic section and one core ERAS-related section (barriers, facilitators, or strategies) were completed. Of the 116 valid respondents, 66 completed the “Barriers” section and 64 completed the “Facilitators” and “Strategies” sections; these participants were included in the corresponding analyses.

The majority of respondents were board-certified specialists (93.9%), and 74.2% reported an oncological subspecialty. Thus, the sample was heavily skewed toward gynecologic oncology specialists (74.2%), limiting generalizability to general gynecology departments. 47.0% worked exclusively in surgical care, while 50.0% reported mixed surgical and conservative duties. Most respondents (75.8%) had more than 10 years of surgical experience, with 12.1% reported 5–10 years. Only 12.1% had less than five years of surgical experience, indicating a sample heavily weighted toward senior clinicians. The predominance of senior clinicians suggests that findings primarily reflect the perspective of experienced specialists rather than trainees or early-career physicians.

Regarding hospital type, 60.6% were employed at secondary care centers, 27.3% at tertiary care institutions (maximum-care), and 12.1% at primary care hospitals. These categories follow standard German definitions of inpatient care, referring respectively to basic (primary care hospitals), intermediate (secondary care hospitals), and specialized care structures (tertiary care hospitals).

ERAS protocols were routinely implemented in 37.9% of institutions, selectively in 16.7%, and not implemented in 45.5%. In this survey, routine ERAS implementation was defined by respondents as applying ERAS measures to most eligible patients, whereas selective implementation referred to procedure- or surgeon-dependent use. These definitions are based on subjective clinician interpretation and do not represent standardized, quantitative thresholds.

ERAS knowledge was assessed as self-reported perceived knowledge: 12.1% felt poorly informed, 37.9% somewhat poorly informed, 34.8% somewhat well-informed, and 15.2% very well-informed. Because these ratings reflect perceived rather than objectively measured knowledge, they may not capture actual protocol understanding. Only 6.1% of clinics reported having a dedicated ERAS consultation service.

63.6% of respondents indicated their department was a certified oncological center. More than half reported that ERAS protocols had not been formally introduced at the time of the survey, while approximately 27% reported implementation within the past five years; only a minority had used ERAS for a longer period.

When asked about collegial participation in ERAS measures, 37.9% indicated that no colleagues implemented ERAS, 39.4% reported that a few colleagues did so, 15.2% reported many, and only 7.6% stated that all colleagues followed ERAS principles.

### 3.2. Barriers to Implementation of ERAS

#### 3.2.1. Distribution of Barriers to the Implementation of ERAS

A total of 66 participants were included in this sub-analysis. To evaluate the internal consistency of the question block addressing “Barriers to implementation of ERAS”, Cronbach’s alpha was calculated, yielding a value of 0.9, which indicates excellent internal reliability [25].

**Figure 1:**
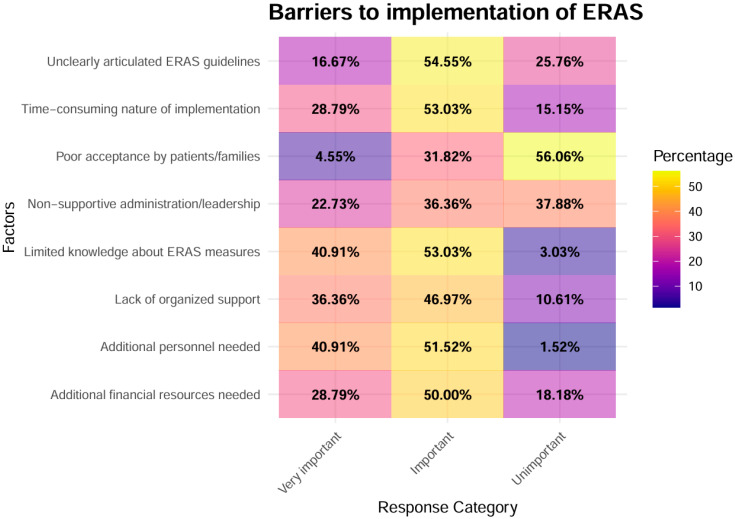
Heatmap of the barriers to ERAS implementation. Response distributions are shown as percentages with color gradients representing response frequency.

Figure 1 shows the distribution of responses to individual barrier items.

Among all assessed barriers, “Limited knowledge about ERAS measures” and “Additional personnel needed” were most frequently rated as “very important” by 40.9% of respondents, and “Lack of organized support” was rated “very important” by 36.36%. These findings suggest that informational and staffing gaps represent the most prominent perceived challenges to ERAS implementation in the surveyed cohort.

#### 3.2.2. Statistical Significance of Both Most Prevalent Very Important Factors

66 respondents evaluated each barrier. To contrast the most frequently endorsed barriers with the remaining items, we used a pooled two-sample test for equality of proportions. Specifically, the three top barriers (3 × 66 = 198 ratings) were compared with the 13 remaining barriers (13 × 66 = 858 ratings). For each group, we calculated the total number of “very important” ratings and divided by the corresponding total number of item-level responses, yielding a pooled “very important” proportion for the top-3 obstacles and a pooled proportion for the remaining obstacles. A one-sided two-sample test for equality of proportions was then conducted, with the alternative hypothesis that the pooled “very important” proportion for the top 3 obstacles was significantly higher than that for the remaining barriers (*p* < 0.001).

Focusing on each of the three most frequently endorsed barriers, “Limited knowledge about ERAS measures among medical staff” was rated as “very important”, which was significantly higher than the pooled proportion of “very important” ratings across all remaining barriers (BH-adjusted *p* < 0.001). Similarly, “Lack of organized support for implementation” also exceeded the pooled proportion for the other barriers (BH-adjusted *p* = 0.0038). Finally, “Additional personnel needed in the clinic” was likewise significantly more often classified as “very important” than the remaining obstacles (BH-adjusted *p* < 0.001). Together, these results underlined that limited ERAS knowledge, insufficient organized support, and additional personnel needs stood out as particularly important barriers compared to the rest of the obstacles evaluated.

#### 3.2.3. Statistical Significance of Both Most Prevalent Unimportant Factors

Regarding barriers, which were frequently perceived as not relevant for ERAS implementation, we examined the two items with the highest proportions of “unimportant” ratings, “Poor acceptance by patients/families” and “Non-supportive administration/leadership”. When these two items were pooled, the proportion of “unimportant” ratings was markedly higher than for the remaining barriers (one-sided test for equality of proportions, *p* < 0.01). In the individual comparisons, “Poor acceptance by patients/families” showed a substantially higher proportion of “unimportant” responses than the other obstacles (BH-adjusted *p* < 0.001). “Non-supportive administration/leadership” was likewise significantly more often classified as unimportant than the remaining barriers (BH-adjusted *p* < 0.01).

### 3.3. Facilitators to Implementation of ERAS

#### 3.3.1. Distribution of Facilitators to Implementation of ERAS

**Figure 2:**
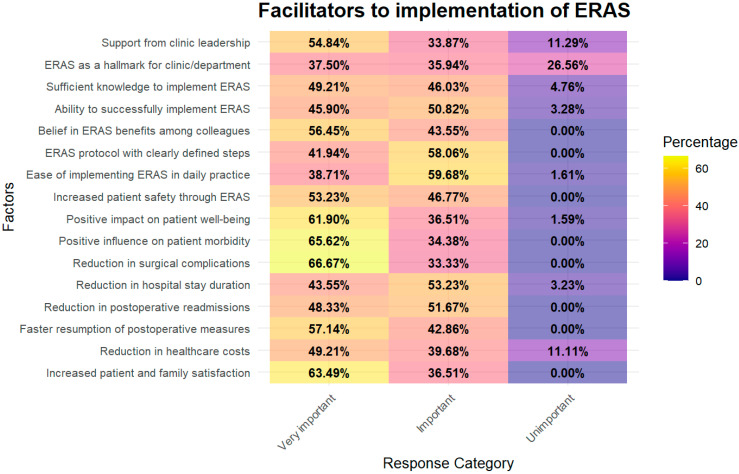
Heatmap showing the percentage distribution of responses regarding perceived facilitators to ERAS implementation, with color gradients representing response frequency.

A total of 64 participants were included in this analysis. To assess the internal consistency of the question block on “Facilitators to ERAS implementation”, Cronbach’s alpha was calculated at 0.784, indicating moderate internal reliability among the questionnaire items [25]. Response distributions are summarized in Figure 2.

#### 3.3.2. Statistical Analysis of the Most Frequently Rated “Very Important” Facilitators to ERAS Implementation

Each of the 16 facilitators was rated by 64 respondents. For the group comparison, we pooled the 4 × 64 = 256 responses for the four most frequently endorsed facilitators and the 12 × 64 = 768 responses for the remaining facilitators. Descriptively, the mean proportion of “very important” ratings (“Positive impact on patient well-being”, “Positive influence on patient morbidity”, “Reduction in surgical complications” and “Increased patient and family satisfaction”) across the four top items was 64.42%. When responses were pooled across the four items (165 of 256 responses), the corresponding pooled proportion was 64.50%, whereas the remaining 12 facilitators reached 48.00% (369 of 768 responses).

To evaluate whether the four most frequently identified facilitators were rated as “very important” more often than the remaining items, we pooled all responses for the top four items (4 × 64 observations) and compared them with the pooled responses for the remaining 12 items (12 × 64 observations) using a two-sample test for equality of proportions. The top four items showed a higher proportion of “very important” ratings (64.50%) than the remaining items (48.00%), and this difference was statistically significant (*p* < 0.001). As this was a single planned comparison, adjustment for multiple testing using the Benjamini–Hochberg procedure did not alter this result.

The individual contribution of each of the four top-rated facilitators was examined by comparing each item separately with the group of all non-top facilitators. All four items showed statistically significant effects before correction for multiple testing: “Positive impact on patient well-being” (*p* = 0.018), “Positive influence on patient morbidity” (*p* = 0.005), “Reduction in surgical complications” (*p* = 0.002), and “Increased patient and family satisfaction” (*p* = 0.010). To account for the four planned comparisons, *p*-values were adjusted using the Benjamini–Hochberg procedure. After this correction, all four facilitators remained statistically significant (BH-adjusted *p*-values ranging from 0.010 to 0.018). These findings indicate that not only was the group of top-rated facilitators clearly distinct from the remaining items overall, but each of the four key facilitators also contributed independently in distinguishing highly rated facilitators from the rest.

#### 3.3.3. Statistical Analysis of the Most Frequently Rated “Unimportant” Facilitators

To examine whether the factors most frequently rated as “unimportant” were distinct from the remaining ERAS facilitators, we first identified the three items with the highest proportion of “unimportant” responses: “ERAS as a hallmark for our clinic/department”, “Support from clinic leadership for the implementation and execution of the ERAS protocol”, “Reduction in healthcare costs through the ERAS protocol”. Each item was rated by 64 respondents. We then pooled responses across these three items (3 × 64 = 192 observations) and compared them with the pooled responses for the remaining ERAS-related factors (13 × 64 = 832 observations) using a two-sample test for equality of proportions. The three most “unimportant” factors showed a substantially higher proportion of “unimportant” ratings compared with the remaining factors, and this difference was statistically significant (*p* < 0.001; *p* = 8.44 × 10^−8^).

In a second step, we examined the individual contribution of each of these three “unimportant” factors by comparing each item separately with the pooled group of all non–top-three “unimportant” ERAS factors. All three items were significantly more often rated as “unimportant” than the remaining facilitators. Specifically, “ERAS as a hallmark for our clinic/department”, “Support from clinic leadership for the implementation and execution of the ERAS protocol”, and “Reduction in healthcare costs through the ERAS protocol” each showed statistically significant differences (all raw *p*-values < 0.01). To control for multiple testing across these three planned comparisons, we applied the Benjamini–Hochberg procedure; all three factors remained statistically significant after adjustment (BH-adjusted *p*-values all <0.01). This indicates that each of the three most “unimportant” facilitators contributed independently to the overall difference between the top “unimportant” group and the remaining ERAS-related factors.

### 3.4. Strategies of ERAS Implementation

#### 3.4.1. Distribution of ERAS Supportive Measures

**Figure 3:**
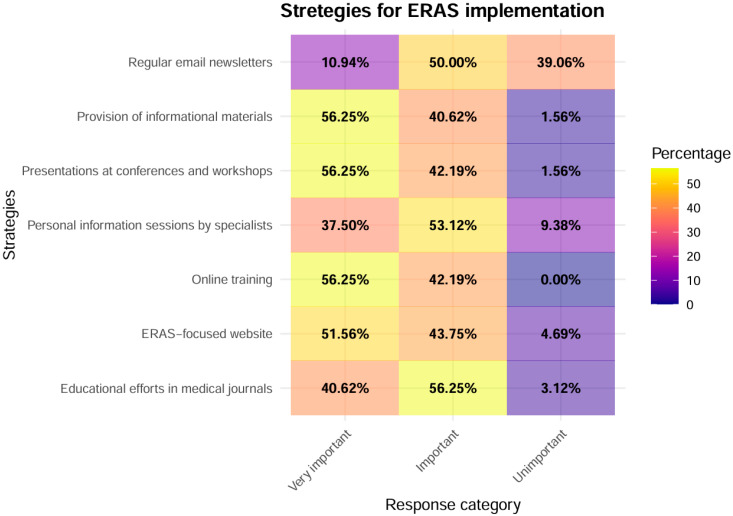
Heatmap of ERAS implementation strategies, with color gradients representing response frequency.

A total of 64 participants were included in the analysis of strategies supporting ERAS implementation. To assess the internal consistency of this question block, Cronbach’s alpha was calculated at 0.68, indicating only fair reliability [25]. Figure 3 illustrates the distribution of responses across all strategy items.

#### 3.4.2. Statistical Analysis of the Most Frequently Rated “Very Important” Strategies

The support strategies most frequently endorsed as “very important” were the “Provision of informational materials for doctors”, “Presentations at conferences and workshops”, “Online training”, and an “ERAS-focused website”. When aggregating all “very important” ratings across the four top strategies and comparing them with all “very important” ratings across the remaining strategies, the difference was statistically significant (*p* < 0.001), indicating a clear preference for these four support measures.

When comparing each support strategy individually against all remaining strategies, the “Provision of informational materials”, “Presentations at conferences and workshops”, and “Online training” were each significantly more likely to be rated as “very important” than the other support measures (all BH-adjusted *p* = 0.033). In contrast, although an “ERAS-focused website” was frequently rated as “very important”, its proportion of “very important” ratings did not differ significantly from the remaining strategies after correction for multiple testing (BH-adjusted *p* = 0.12).

#### 3.4.3. Statistical Analysis of the Most Frequently Rated “Unimportant” Strategies

“Regular email newsletters” were markedly more often rated as “unimportant” than the other support strategies. The proportion of “unimportant” responses for “Regular email newsletters” was approximately 39%, compared with an average of approximately 3% across the remaining strategies (*p* < 0.001). A one-sided test for equality of proportions confirmed that regular email newsletters were significantly more likely to be rated as unimportant than the remaining support measures (*p* < 0.001).

### 3.5. Perceived Influence of ERAS Program on Professional Life

#### 3.5.1. Distribution of ERAS Program Influence on Professional Life

A total of 64 participants were included in this analysis. The distribution of responses regarding the influence of ERAS on daily professional routines revealed that 65.62% of respondents indicated that ERAS measures had a positive impact on their professional life, while 34.38 reported no influence. None of the respondents reported a negative influence on their professional life.

#### 3.5.2. Influence of Certification Status and ERAS Knowledge on Professional Life

A logistic regression analysis was conducted to assess whether being well-informed about ERAS procedures or working in a DKG-certified center was associated with perceived improvements in professional life. Respondents who felt well-informed about ERAS had significantly higher odds of reporting a positive impact on their professional life (OR = 9.00, 95% CI: 2.76–35.95, *p* = 0.001, R^2^ = 0.278). Being employed in a DKG-certified center, however, did not show a statistically significant association with perceived professional benefit (OR = 1.38, 95% CI: 0.47–4.03, *p* = 0.549, R^2^ = 0.004).

## 4. Discussion

This study aimed to identify the key barriers, facilitators, and implementation strategies influencing ERAS adoption in German gynecological departments. Our findings show that limited staff knowledge and insufficient personnel were the most commonly perceived barriers, whereas patient-centered benefits and interactive educational approaches were the most influential facilitators. These results align with prior research in other surgical fields but also highlight unique aspects of the German context, particularly regarding perceptions of administrative support and the substantial influence of ERAS on professional satisfaction.

An important contextual consideration is the composition of the study population. The majority of respondents reported a gynecologic oncology subspecialty and more than ten years of surgical experience. Consequently, the findings primarily reflect the perceptions of senior, oncology-focused clinicians. Implementation dynamics, perceived barriers, and facilitators may differ in general gynecology settings or among residents and early-career physicians, who may experience ERAS protocols from different operational and hierarchical perspectives. The present results should therefore be interpreted within this specific professional context and not generalized to all German gynecologic departments.

Effective implementation of new clinical protocols, such as ERAS, requires a clear and proper understanding of the key barriers and facilitators that are usually encountered in daily practice. Previous studies have identified several ERAS implementation barriers, including patient-level factors such as language or cost barriers, and institutional-level challenges. In our study, 56.1% of respondents did not consider patient or family acceptance as a barrier, although nearly 45% still viewed it as a potential concern, consistent with prior reports on patient preference as an implementation obstacle [26]. An international study showed that only 20% of participants considered patients as a barrier, suggesting approximately 80% did not prioritize it [12], a lower frequency than the 36.3% which considered poor acceptance by patients/families as a “very important” or important” barrier, as revealed by this study. This difference may reflect cultural or structural characteristics of patient communication in Germany, where perioperative routines and expectations may differ from those in countries reporting higher perceived resistance. It also suggests the need to better understand how educational materials, shared decision-making practices, or preoperative counseling influence German patients’ readiness to engage with ERAS components. Nevertheless, patient education about the benefits of ERAS should be included as a key element in order to successfully implement ERAS measures [27], as there are reports citing patient resistance as barriers to implementation [11,12,13,14,15,16,17,18,19,20,21,22,23,24,25,26].

Among institutional barriers, limited staff knowledge of ERAS measures emerged as the most consistently rated barrier (40.9% “very important”, 53.0% “important”), echoing earlier findings that education deficits impede ERAS adoption [10]. Lack of knowledge was the primary barrier to ERAS adoption among non-users, with over half unaware of the protocol and reporting deficits in information and training [17]. Evidence from gastrointestinal surgery further supports the view that ERAS implementation is shaped by complex organizational and cultural dynamics. In these settings, uncertainty about ERAS safety (reported by 85.7% of staff) and the perception that ERAS is largely physician-driven (76.2%) were major obstacles. More than half of personnel described insufficient multidisciplinary collaboration, and 22% reported inadequate interdepartmental coordination. Additional concerns, such as variability in patient conditions (88.1%), fear of postoperative complications leading to conflict (61.9%), and difficulty achieving patient engagement (26%), mirror several of the barriers observed in our cohort [28]. Gramlich et al. emphasize the importance of framing ERAS education around patient-centered benefits [10]. Also, Tobiano et al. showed that education was the most frequently implemented strategy used in the implementation of practice change programs and was key to building capability in ERAS implementation [27].

Insufficient personnel resources were frequently identified as an important barrier. “Additional personnel needed” was rated “very important” by 40.9% and “important” by 51.5%. A study conducted in China revealed that all participants regarded the shortage of human resources as a barrier to ERAS implementation [13]. These results were reproduced by international studies, which showed that environmental context and funding, including staff shortages, as well as lack of knowledge, ultimately led to barriers to ERAS implementation [17,29,30,31,32]. However, staffing shortages represent a broader structural challenge within the healthcare system and are not specific to ERAS implementation. While relevant, such macro-level capacity constraints may be less directly modifiable at the departmental level.

Other resource-related items, such as “additional financial resources”, “time-consuming implementation,” and “lack of organized support”, were less consistently rated but remained relevant. These trends align with international findings on financial and administrative hurdles in ERAS implementation [26,27,28,29,30,31,32,33].

With regard to the economic burden, ERAS implementation may require time and upfront financial support, such as protocol development and hiring supporting staff [11]. Additionally, initial cost investment is often not covered by insurance and reimbursement systems [14]. Although initial investment and limited support have traditionally been reported as major obstacles, stakeholders increasingly balance these concerns against the substantial long-term financial benefits documented in the literature [34,35]. In the long run, ERAS has been shown to generate substantial financial benefits, with return-on-investment models demonstrating up to $7.31 saved per dollar spent, especially by minimizing hospitalization and medication costs, and studies in gynecologic oncology reporting cost savings exceeding $2000 per patient [4,36,37].

Contrary to much of the international literature, which frequently identifies lack of administrative or leadership support as a major barrier to ERAS implementation, our respondents rated this factor among the least “very important” obstacles (22.7% of respondents). In the previous international literature, lack of administrative and institutional support, such as absent leadership engagement, insufficient incentives, and poor coordination, was consistently identified as a major barrier to ERAS implementation, limiting healthcare provider motivation and slowing adoption despite clinical enthusiasm [11,13,20,38]. However, it is important to distinguish between perceived leadership engagement and objectively measurable structural or financial support. The present survey assessed clinicians’ subjective perceptions of whether administration or departmental leadership was supportive, rather than independently verified institutional resource allocation, staffing approval processes, or budgetary commitment.

It is therefore possible that respondents experience leadership as supportive in principle, while structural constraints such as limited funding, staffing ceilings, or reimbursement policies remain unchanged. Alternatively, structural support mechanisms may not be fully visible to frontline clinicians. This distinction may help explain the apparent divergence from prior studies that evaluated administrative support more broadly at organizational or system levels.

Consequently, our findings should not be interpreted as evidence that institutional or financial support is unimportant for ERAS implementation, but rather that the lack of perceived leadership engagement was not considered the most immediate barrier within this specific respondent cohort.

It is also important to consider the structural characteristics of the German healthcare system during the survey: gynecologic departments in Germany were permitted to perform oncologic surgery even if they were not certified oncologic centers and even if individual surgeons did not hold formal subspecialization in gynecologic oncology. This structural flexibility creates considerable heterogeneity in organizational conditions, team expertise, and perioperative routines, which may partly explain why certain implementation barriers, such as leadership support or institutional incentives, were perceived differently in our cohort compared to findings from more centralized or subspecialty-driven healthcare systems.

Additionally, no significant differences were detected between DKG-certified gyneco-oncological and non-certified centers, nor between respondents who felt well-informed versus poorly informed about ERAS measures. This suggests that perceptions of fundamental barriers may be systemic rather than dependent on institutional certification status or individual knowledge levels.

On the other hand, leadership support, as well as champion behavior and coherence, were seen as important measures to overcome barriers [11,20]. In order to overcome institutional barriers, targeted tools that engage hospital leadership, such as clear implementation frameworks, cost–benefit analyses, and defined incentives, are essential to securing administrative support and thus will result in sustainable ERAS adoption [10,20,21,29,30,39].

The most important facilitators to ERAS implementation, with over 60% of respondents rating “very important,” were the patient-centered outcomes, such as positive effects on patients’ morbidity and the increase in well-being and satisfaction, as well as reduction in surgical complications. Importantly, all four patient-focused facilitators remained statistically significant in individual analyses after correction for multiple testing, confirming that each independently contributed to the overall pattern. These findings are in line with several other publications that showed that increased patient satisfaction and decreased morbidity were the key drivers for the implementation of ERAS protocols [12,38,40,41]. These findings suggest that proper education about the various benefits of ERAS would increase willingness to follow the protocol. These strong directional effects reinforce the centrality of patient outcomes in driving clinician motivation and willingness to adopt ERAS principles. Nevertheless, although adjustments for multiple testing were applied using the Benjamini–Hochberg procedure, the relatively large number of parallel comparisons increases the residual risk of false-positive findings. This concern is amplified by the limited effective sample size, as small datasets are more susceptible to random variation. Consequently, statistically significant associations should be interpreted as exploratory patterns rather than definitive evidence of stable effects.

Moreover, a protocol with clearly defined steps was regarded by the study population in 40.6% and 56.2% as “very important” and “important”, respectively. Clearly defined, procedure-specific ERAS protocols, standardized across departments and included in electronic health records, are essential tools to support consistent, evidence-based care and improve adherence among multidisciplinary teams [10,27,28,31,32,39,41,42].

High-impact implementation strategies in this study included the provision of informational materials, participation in workshops and conferences, and online training, each of which was rated as “very important” by more than 50% of respondents. Consistent with prior research, these interactive, active-learning formats were viewed as more effective than passive approaches such as journal articles or email newsletters [10,21]. This preference aligns with established evidence showing that active learning stimulates critical thinking, reinforces knowledge retention, and facilitates knowledge transfer into clinical practice [43,44]. The emphasis on interactive, team-based formats corresponds to findings from Pache et al., who identified continuous staff education, a dedicated ERAS coordinator, and regular interdisciplinary team meetings as core success factors for ERAS implementation [12]. Similarly, previous studies highlight the importance of in-person education, including workshops and structured team-based sessions, for improving the uptake of new healthcare measures [20,26,45].

An expert panel has also recommended delivering the ERAS curriculum through multidisciplinary, team-based training, combining both face-to-face and online methods, integrating ERAS teaching into undergraduate medical education, and using ongoing audit and feedback to assess and reinforce effectiveness [21]. This aligns with evidence from Cochran et al., who demonstrated that although surgeons often assume primary responsibility for ERAS implementation, nurses, anesthesiologists, and advanced practice providers play essential roles, underscoring the multidisciplinary nature of effective ERAS delivery [16]. Furthermore, continuous staff education and setting realistic expectations are critical for sustaining ERAS over time, particularly in environments with high staff turnover, where repetitive training, mentorship, and outcome-based auditing remain just as crucial as initial implementation efforts [9,12].

Although personal information sessions were ranked lower than other strategies, they remain valuable for clarifying responsibilities, supporting team-building, and illustrating clinical or financial benefits through real-case examples and audit feedback [12,18,21,46]. Participants strongly favored approaches that enable interaction, discussion, and peer learning, reinforcing the importance of team communication and shared decision-making in establishing consistent ERAS pathways.

The survey also found that ERAS-focused websites were rated as “very important” by over 50% of participants. These platforms offer open-access, evidence-based guidelines, interactive audit tools, and downloadable educational materials, which play a critical role in supporting implementation and promoting standardized, high-quality perioperative care. By providing accessible and regularly updated resources, ERAS-focused websites facilitate the global dissemination of specialized knowledge and improve consistency across institutions [20,21,47,48].

Finally, participants rated email newsletters and medical journal publications as among the least important strategies, reflecting established concerns about accessibility, time constraints, and passive information delivery. Nevertheless, manually generated paper reminders have been shown to improve professional practice and adherence to clinical recommendations, as demonstrated in a recent Cochrane Review [49], suggesting that simple, low-tech interventions may still contribute meaningfully when integrated thoughtfully into a broader, active-learning–based implementation strategy.

These findings together suggest that implementation strategies should emphasize not only the clinical benefits of ERAS but also its potential to improve workflow efficiency, reduce professional stress, and enhance team cohesion. Framing ERAS as an opportunity for professional development, or establishing “ERAS Ambassador” roles that highlight these benefits, may increase clinician engagement and accelerate adoption.

In this exploratory analysis, self-reported higher ERAS familiarity was associated with reporting a positive professional impact (OR = 9.0, *p* = 0.001). This shows a dual benefit: ERAS improves patient outcomes and enhances staff satisfaction. This finding suggests that perceived knowledge and familiarity with ERAS protocols, rather than institutional certification status, play a more influential role in shaping positive professional experiences than formal institutional certification alone. The association between familiarity with ERAS procedures and improved professional satisfaction may arise from several mechanisms: more predictable postoperative trajectories, fewer complications, clearer protocols that streamline decision-making, and improved interdisciplinary coordination. Together, these factors may create a more structured and less stressful work environment, which may explain why well-informed clinicians perceived ERAS as enhancing their daily practice. These professional advantages represent an underused but potentially powerful strategy for motivating ERAS adoption, which should be emphasized in future implementation campaigns to strengthen motivation and support institutional change, as job satisfaction was previously positively associated with maintaining personal balance, reducing professional stress, and subsequently improving medical and financial outcomes in patient care [50]. However, ERAS familiarity in this study was assessed as self-reported perceived knowledge rather than objectively measured competence or verified protocol adherence. Consequently, this variable reflects subjective confidence in ERAS understanding rather than actual implementation fidelity. It is therefore possible that the observed association relates to professional confidence, engagement, or personal interest in ERAS rather than to objectively higher-quality protocol execution. These findings should not be interpreted as evidence that greater ERAS knowledge causally improves professional satisfaction or clinical performance, but rather as an association between perceived familiarity and reported professional experience within this respondent cohort. Additionally, given the limited effective sample size and wide confidence intervals, this association should be interpreted cautiously. The cross-sectional design does not permit conclusions regarding directionality or causality.

To translate the identified barriers into actionable strategies for the German context, several system-level recommendations emerge from our findings. First, addressing the knowledge gap would benefit from establishing a standardized national ERAS education curriculum for gynecology, ideally delivered through a centralized, accredited online platform and supplemented by interactive workshops. Such a curriculum could draw upon existing international ERAS frameworks while incorporating German-specific organizational and regulatory structures.

To ensure feasibility at the institutional level, such educational initiatives could be coordinated by a designated ERAS lead or “ERAS champion” within each department, supported by a small multidisciplinary core team including representatives from surgery, anesthesiology, and nursing. Clearly defined leadership roles may enhance accountability, continuity, and interdisciplinary alignment. Structured onboarding modules for new staff and regular case-based workshops may facilitate sustained knowledge transfer, particularly in settings with frequent personnel turnover.

Second, to mitigate personnel shortages, which were identified as a major obstacle, future research should explore innovative staffing models, including the introduction of dedicated ERAS coordinators or role-adapted task-sharing between nursing, surgical, and anesthesiology teams.

From an implementation perspective, barriers may be categorized into structural (e.g., staffing capacity), organizational (e.g., lack of structured support), and individual-level factors (e.g., knowledge and familiarity). While structural barriers require long-term policy and funding interventions, organizational and individual-level barriers may be more immediately addressable through targeted implementation strategies. Our findings suggest that educational initiatives and standardized coordination mechanisms may represent the most feasible starting points for improving ERAS uptake.

To support sustainability, departments could implement regular audit-and-feedback cycles using predefined performance indicators, such as adherence to key ERAS components (e.g., early mobilization, early oral intake), postoperative complication rates, length of hospital stay, and patient-reported recovery measures. Periodic interdisciplinary review meetings may facilitate identification of workflow bottlenecks and reinforce adherence through data-driven feedback.

Conducting German-specific cost–benefit analyses of ERAS-related staffing models would further strengthen the economic rationale for targeted resource allocation. Evidence from multiple German surgical disciplines already demonstrates that ERAS implementation is associated with net financial benefits within the German DRG system, despite higher upfront personnel or organizational investments. In colorectal surgery, ERAS adoption led to a marked reduction in overall treatment costs and increased contribution margins, even though DRG valuation ratios decreased, confirming economic feasibility under routine reimbursement conditions [51]. Similarly, a German micro-costing analysis in minimally invasive cardiac surgery demonstrated significant per-patient cost savings driven primarily by reduced length of stay and ICU utilization, despite increased physiotherapy-related expenditures [52]. In liver surgery, ERAS implementation was shown to offset the costs of additional workforce and structured perioperative care by substantially reducing postoperative complications, which were identified as the major drivers of financial loss under DRG conditions [53]. Taken together, these German data suggest that investments in dedicated ERAS personnel and structured implementation strategies are likely to be cost-effective rather than cost-burdensome, supporting the need for specialty-specific economic evaluations in gynecologic surgery to facilitate sustainable ERAS adoption.

Finally, the finding that administrative or leadership support was not perceived as a major barrier presents a unique opportunity within the German gynecological landscape. Clinicians may therefore be well positioned to proactively engage institutional leadership by presenting performance data, workflow improvements, and staffing needs aligned with ERAS implementation. Such strategically aligned collaboration could accelerate system-level uptake and ensure sustainable integration of ERAS pathways, as outlined in established implementation frameworks.

### Strengths and Limitations

This study offers several notable strengths. It employed robust statistical methods, including tests for internal consistency (Cronbach’s alpha), equality of proportions, and logistic regression analyses, in order to enhance the reliability and interpretability of the findings. The questionnaire was carefully developed following a thorough literature review and encompassed key domains of ERAS implementation, such as barriers, facilitators, and strategies. As a novel contribution, this study offers a specialty- and region-specific perspective on ERAS implementation. Importantly, the study not only assesses procedural adoption but also explores the perceived influence of ERAS on professional life. As such, it offers a novel insight into its potential to enhance staff satisfaction, a to date underexamined but potentially impactful facilitator of implementation in the future.

The relatively small number of participants (N = 64–66 per subsection) limits statistical power and may have prevented the identification of less frequently perceived barriers or facilitators. As a result, true effects may remain undetected (risk of Type II error), and findings should be interpreted as exploratory rather than definitive. As such, the findings must be interpreted as preliminary and may not be generalizable to all German gynecological departments, and the results may not be directly applicable to other countries, healthcare systems, or surgical specialties.

Because participation was voluntary and distributed via email, respondents were likely more interested or engaged in ERAS than non-respondents, which introduces the possibility of selection bias. This self-selection may have led to overestimation of perceived benefits or underestimation of resistance. Future studies should employ multi-modal recruitment, e.g., departmental outreach, professional society networks, or conference-based enrolment, to reduce this bias. Additionally, all results are based on self-reported perceptions, which may be affected by recall bias or social desirability, although anonymity may have helped to mitigate these factors. Moreover, several key variables, particularly perceived knowledge of ERAS, were based on self-report and may not reflect objectively measured knowledge or adherence. Also, aggregation bias is possible because individual respondents reported departmental-level information. Future studies should aim to collect data from multiple team members within each institution or incorporate institutional-level documentation to better align perceptions with organizational reality.

Moreover, several subgroup analyses, including comparisons by certification status, were based on relatively small subgroup sizes. Consequently, effect estimates are associated with wide confidence intervals and should be considered statistically fragile. Small changes in response distribution could materially alter the observed associations. These findings should therefore be interpreted as exploratory and hypothesis-generating rather than confirmatory.

A further methodological limitation relates to section-specific non-response and declining completion rates across the survey. While 116 responses met the inclusion criteria, only 66 respondents completed the barriers section, and 64 completed the facilitators and strategies sections. This reduction in effective sample size introduces the possibility of attrition bias if respondents who completed later sections differed systematically from those who discontinued participation. As a result, reported proportions and regression estimates may reflect the perceptions of a more engaged or ERAS-interested subgroup rather than the broader initial respondent pool. The potential impact of this selective completion limits the stability and generalizability of section-specific findings.

While most question blocks demonstrated strong internal consistency, the section addressing implementation strategies showed only fair reliability (Cronbach’s alpha = 0.68), potentially limiting the robustness of specific item-level conclusions. This section likely reflects substantive heterogeneity among the listed strategies (e.g., printed materials, digital tools, workshops, and newsletters), and the question block may represent conceptually distinct modalities rather than a unified construct. Future refinements to the questionnaire should consider subdividing this block into more coherent categories to improve reliability, by reorganizing these items into more coherent sub-categories to improve construct validity, e.g.,: educational formats (e.g., workshops, webinars, interactive training), digital resources (e.g., ERAS-focused websites, online modules, email materials) and personalized support (e.g., dedicated ERAS coordinators, peer-to-peer mentoring). Such thematic grouping would allow clearer interpretation of response patterns and more robust assessment of distinct implementation strategies.

Additionally, the cross-sectional study design limits causal inference and captures only a snapshot in time, preventing assessment of evolving practices. Finally, while responses were collected at the individual level, some questions addressed institutional practices, introducing a risk of aggregation bias when extrapolating departmental behaviors from individual perceptions.

## 5. Conclusions

Taken together, this exploratory, cross-sectional survey provides context-specific insights into ERAS implementation within German gynecological departments, offering a perspective that complements international research predominantly focused on colorectal surgery, other surgical specialties, or non-European healthcare systems.

This study provides insight into the current status of ERAS implementation in German gynecological departments. Adoption remains inconsistent, with limited staff knowledge and insufficient personnel identified as the most significant barriers. Patient-centered benefits, including improvements in well-being, morbidity, and satisfaction, were the strongest facilitators, while interactive and accessible educational strategies were perceived as most helpful for supporting implementation.

Given the modest sample size, incomplete section responses, and predominance of senior and oncology-focused respondents, these findings should not be interpreted as representative of all German gynecologic departments. Rather, they reflect perception-based patterns within a self-selected cohort and should be considered hypothesis-generating.

A novel and important finding is the strong association between ERAS knowledge and improved professional satisfaction among clinicians. This suggests that ERAS may not only improve patient outcomes but also enhance provider well-being, workflow efficiency, and perceived professional satisfaction, a dimension rarely highlighted in previous ERAS research. Highlighting these professional benefits may represent an underused but powerful strategy for motivating adoption.

To strengthen ERAS uptake, implementation efforts should prioritize improving staff knowledge, ensuring sufficient human resources, and communicating both the clinical and professional advantages of ERAS in a structured, multidisciplinary manner.

These results suggest that modifiable factors such as structured education, standardized protocols, and interdisciplinary coordination may represent potential areas for further investigation in efforts to support ERAS uptake.

## Data Availability

The data were collected anonymously and without personal identifiers. Informed consent did not include public data deposition; therefore, the dataset is available from the corresponding author upon reasonable request and with appropriate ethical considerations.

## Supplementary Materials

The following supporting information can be downloaded at: https://www.mdpi.com/article/10.3390/healthcare14050682/s1. Questionnaire “Implementation of Enhanced Recovery after Surgery in Germany: An anonymous cross-sectional study”.
