# Supplementary material for: Paving the Way for ERAS in German Gynecologic and Gynecologic Oncology Departments: Insights into Barriers, Facilitators and Practical Strategies
Source: Healthcare (Basel). 2026 Mar 8;14(5):682. doi: 10.3390/healthcare14050682 (PMC12984466; doi:10.3390/healthcare14050682)
Supplement: Supplementary file 1 [file healthcare-14-00682-s001.zip › healthcare-4118599-supplementary.pdf]

Questionnaire "Implementation of Enhanced Recovery after Surgery in Germany: An anonymous cross-sectional study"

Welcome to this survey! Please read the following information carefully and then provide your consent to proceed.

Purpose of this Study:
This study's objective is to evaluate the implementation and execution of ERAS (Enhanced Recovery After Surgery) protocols in gynecological oncology in Germany.

Eligibility Criteria:
All German-speaking physicians specialized in gynecology are eligible to participate in this study. If you do not meet these criteria, you will be notified during the first part of the survey, and the survey will be terminated.

Ethics and Anonymity:
This survey includes questions regarding the implementation and your evaluation of ERAS measures but does not collect personal, identifiable data. All data is captured anonymously, ensuring that your responses cannot be traced back to you.

Important Information:
The survey takes approximately 5-10 minutes. You may discontinue the survey at any time without stating the reasons. Please read each question carefully and answer as accurately as possible.

Above the response field, you will find line-by-line additional information or instructions.

Good ethical research practice requires that participants in empirical studies explicitly and clearly consent to participation and the publication of anonymized data.

Confirmation of participation (mandatory to proceed to the questionnaire):
I hereby confirm that I am at least 18 years old and have read and understood the consent form.
I have read and understood the study information and agree to voluntarily participate in the online survey "Application of ERAS (Enhanced Recovery After Surgery) in Clinical Practice in the German-speaking Region: An Anonymous Web-based Cross-sectional Study."

Please specify your current status in specialist medical training (single choice):

- Resident in Gynecology and Obstetrics
- Specialist in Gynecology and Obstetrics

Do you specialize in gynecological oncology (single choice):

- No
- Yes

Are you primarily engaged in surgical or conservative practice (single choice):

- Conservative

- Surgical
- Both

How long have you been practicing surgery in the field of gynecological oncology? Personal surgical experience (single choice):

- 0 to under 5 years
- 5 to under 10 years
- Over 10 years

What type of care does your clinic provide (single choice):

- Primary care provider
- Secondary care provider
- Tertiary care provider

Is your clinic a certified center (No, Yes):

- DKG (German Cancer Society)
- ESGO (European Society of Gynecologic Oncology)
- ERAS (Enhanced Recovery After Surgery)

Do you implement ERAS in your clinic?

- No
- Yes
- In selected cases

Even if you do not implement the ERAS concept in your clinic/department, it is advisable to answer the following questions, as certain steps might still be implemented in clinical practice.

Do you feel well-informed about the ERAS concept?

- Poor
- Somewhat poor
- Somewhat well
- Very well

Does your hospital have a dedicated ERAS consultation service?

- No
- Yes

When was the ERAS concept introduced in your clinic?

- Not yet introduced
- Less than 1 year
- 1 to less than 2 years
- 2 to less than 5 years
- 5 to less than 10 years

- Over 10 years

Do your colleagues implement ERAS?

- None
- Few
- Many
- All

During which oncological surgical procedures do you implement ERAS?

(never, rarely, often, always)

- During laparotomies
- During laparoscopies

What topics do you discuss in the context of prehabilitation?

(never, rarely, often, always)

- Recommendation for aerobic and resistance training to enhance physical functionality, body composition, and cardiorespiratory health
- Advice on specific functional exercises aimed at reducing or preventing functional impairments
- Nutritional counseling to promote recovery post-operation and address malnutrition associated with the disease or its treatment
- Psychological support to reduce stress, promote behavioral changes, and improve overall well-being
- Psychosocial counseling
- Counseling on resilience-enhancing measures
- Fragility screening in older or fragile patients
- Recommendation to quit smoking (only for smokers)
- Recommendation to reduce alcohol consumption (for patients who regularly consume alcohol)
- Evaluation of anemia and possible initiation of iron supplementation
- Evaluation of pre-existing diabetes and possible optimization of metabolism (e.g., by GP, endocrinologist)
- Optimization of therapy for pre-existing conditions (e.g., COPD) by GP or other specialists

How do you conduct preoperative fluid restriction/fasting or food restriction/fasting?

(2 hours pre-op, 6 hours pre-op, 6-8 hours pre-op, 24 hours pre-op)

- Fluid restriction (clear liquids) from:
- Food restriction (solid food) from:

Preoperative Bowel Preparation: For which procedures is preoperative bowel preparation performed?

(never, rarely, often, always)

- Planned Laparotomy:
- Planned Laparoscopy:
- Likelihood of Surgical Intervention on the Bowel:
- Debulking for Ovarian Cancer:
- Planned Exenteration:
- As Desired by the Multidisciplinary Team:
- Anticipated Complicated/Difficult Surgical Conditions (e.g., Obesity, Adhesions):

How do you normally conduct bowel preparation? (Single choice)

- I do not perform bowel preparation.
- Mechanical only (e.g., enema, laxatives).
- Oral antibiotics only.
- Oral antibiotics and mechanical preparation.
- Other methods.

Do you recommend a carbohydrate-rich diet preoperatively (e.g., carbohydrate-containing drinks)?

- Never
- Rarely
- Often
- Always

Do you conduct thrombosis prophylaxis?

(never, rarely, often, always, only for high risk of thromboembolic events)

- Preoperatively:
- Intraoperatively:
- Postoperatively:

How do you typically conduct thrombosis prophylaxis?

(Unfractionated heparin, Low molecular weight heparin, Oral anticoagulants, Mechanical compression, Dual prophylaxis: heparin and mechanical compression)

- Preoperatively:
- Intraoperatively:
- Postoperatively:

How long do you continue postoperative thrombosis prophylaxis based on the risk of thromboembolic events?

(1 week, 2 weeks, 3 weeks, at least 4 weeks)

- Low risk:
- Moderate to high risk:
- High risk:

What risk factors for thromboembolic events do you consider important for extending postoperative anticoagulation in gynecological-oncological patients?

(very unimportant, unimportant, important, very important)

- Following open surgery (laparotomy):
- Following minimally invasive surgery (laparoscopy):
- High tumor stage:
- Obesity:
- Prolonged immobilization:
- Long duration of surgery:
- Advanced age:
- Thromboembolic events in personal medical history:
- Thromboembolic events in family medical history:

Do you use preoperative sedatives and/or anxiolytics?

- Never
- Sometimes
- Always

How is intraoperative fluid management conducted?

- No fluid management is conducted
- At the discretion of the anesthesia team
- According to invasive targeted monitoring (e.g., esophageal doppler)
- According to non-invasive targeted monitoring (e.g., blood pressure, urine output)
- Method is unknown

Is core body temperature monitored to maintain it as stable as possible intraoperatively?

- Never
- Sometimes
- Always
- Unknown

Do you implement a multimodal pain management strategy as part of the ERAS program?

- Never
- Always
- Sometimes

Which methods do you apply in multimodal pain management?

(never, rarely, often, always)

- Intraoperatively, short-acting anesthetics:
- Short-acting opioids:
- Perioperative regional pain management using a Transversus Abdominis Plane (TAP) Block:
- Intra-/perioperative infiltration of incision sites (subcutaneous fat tissue, fascia) with a local anesthetic (Bupivacaine, liposomal Bupivacaine):
- Postoperatively, NSAIDs, prophylactically set:
- Postoperatively, NSAIDs as additional medication for pain exacerbation:
- PCA pump (patient-controlled analgesia):
- Oral opioids (aiming for the lowest total dose):
- Selective COX-2 inhibitors:
- Gabapentin:
- Restrictive algorithm for opioid prescription upon discharge:

When do you typically remove the following foreign bodies postoperatively?

(At the end of surgery, Within 6 hours postoperatively, On the 1st postoperative day, From the 2nd postoperative day)

- Gastric tube:
- Urinary catheter:
- Central venous catheter:
- Intra-abdominal drainage:
- Suprafascial drainage:

When do you typically pause the postoperative administration of intravenous fluids?

- Within 12 hours after surgery
- 12–24 hours after surgery
- After 24 hours
- As soon as the patient can adequately intake fluids orally

What measures do you take to prevent postoperative ileus?

(never, rarely, often, always)

- Chewing gum:
- Prokinetics (e.g., Metoclopramide):
- Laxatives (e.g., Bisacodyl):
- μ-Opioid antagonist (e.g., Naloxegol, Naloxone, Naldemedine, Methylnaltrexone):

How do you manage the postoperative diet?

(Liquid diet on the day of surgery, Light diet on the day of surgery, Tolerance diet on the day of surgery)

- After surgery without bowel resection:
- After surgery with bowel resection:

How do you manage postoperative mobilization (average start time)?

- On the day of surgery
- From the 1st postoperative day
- From the 2nd postoperative day

What measures do you take to prevent postoperative infection?

(never, rarely, often, always)

- Preoperative skin preparation, e.g., showering with chlorhexidine-containing shower gel/soap:
- Preoperative antibiotic prophylaxis:
- Repetition of intraoperative antibiotic prophylaxis (e.g., for long surgery duration, high blood loss):
- Postoperative antibiotic prophylaxis:
- Regulation of core body temperature (pre-/intra-/postoperatively) to maintain normothermia:
- Avoidance of foreign bodies (e.g., drainages):
- Active control of postoperative hyperglycemia:

What factors do you consider as obstacles in implementing ERAS measures?

(very important factor, important factor, unimportant factor)

- Non-supportive administration/management/clinic leadership:
- Limited knowledge about ERAS measures among medical staff:
- Unclearly articulated ERAS guidelines:
- Time-consuming nature of implementing the ERAS program:
- Lack of organized support for implementation:
- Additional personnel needed in the clinic:
- Additional financial resources needed in the clinic:
- Poor acceptance of ERAS measures by patients and patient families:

What factors do you consider as motivators for implementing ERAS measures?

(very important factor, important factor, unimportant factor)

- Support from clinic leadership for the implementation and execution of the ERAS protocol:
- ERAS as a hallmark for our clinic/department:
- Having sufficient knowledge to implement the ERAS protocol in clinical practice:
- Being able to successfully implement the ERAS protocol:
- Belief in the benefits of the ERAS protocol among colleagues from various disciplines involved in its execution (e.g., anesthesiologists):
- ERAS protocol consisting of several clearly defined steps/units:

- Ease of implementing the ERAS protocol in daily clinical practice:
- Increased safety for patients through the ERAS protocol:
- Positive impact on the well-being of gynecological-oncological patients through the ERAS protocol:
- Positive influence of the ERAS protocol on the morbidity of gynecological-oncological patients:
- Reduction in complications during the surgical treatment of gynecological-oncological patients through the ERAS protocol:
- Reduction in hospital stay duration through the ERAS protocol:
- Reduction in postoperative readmissions to the hospital through the ERAS protocol:
- Faster resumption of postoperative planned therapeutic measures (e.g., chemotherapy) through the ERAS protocol:
- Reduction in healthcare costs through the ERAS protocol:
- Increased satisfaction among patients and patient families through the ERAS protocol:

How can doctors and clinics be supported in implementing and adhering to the ERAS concept?

(very important, important, unimportant)

- Generous provision of informational materials for doctors:
- Presentations at conferences and workshops:
- Online training:
- ERAS-focused website:
- Regular email newsletters:
- Educational efforts on the benefits and rationale of ERAS in medical journals:
- Personal information sessions by specialists:
- How does the ERAS program influence your professional daily routine?

The ERAS program influences my professional everyday life (negative, positive, no influence)
